# Molecular characterization of cDNA coding for 33.5 and 41 kDa oocyst and sporocyst proteins that are differentially regulated in different strains of *Eimeria maxima*

**DOI:** 10.3389/fvets.2024.1445646

**Published:** 2024-09-27

**Authors:** Mark C. Jenkins, Carolyn Parker, Andrew Jansen, Marianne Dias Papadopoulos, Matthew S. Tucker

**Affiliations:** ^1^Animal Parasitic Diseases Laboratory, NEA, BARC, ARS, USDA, Beltsville, MD, United States; ^2^Electron and Confocal Microscopy Unit, SEL, NEA, BARC, ARS, USDA, Beltsville, MD, United States

**Keywords:** *Eimeria maxima*, recombinant protein, 2D gel, immunofluorescence, *Eimeria*, RT-PCR, EMWEY 23530, EMWEY 48910

## Abstract

*Eimeria maxima* (APU1 and APU2) differ in virulence for chickens, due in part to the greater fecundity of the former. In a previous study, RNA-seq was used to identify a transcripts upregulated in *E. maxima* APU1 compared to *E. maxima* APU2. In this study, 2 of these upregulated genes (*EMWEY 23530* and *EMWEY 48910*) were characterized by first confirming upregulation using quantitative RT-PCR. For both *EMWEY 23530* and *EMWEY 48910*, RNA transcription was fairly consistent during sporulation. The extent of differential expression was about 2-fold log_2_ higher in APU-1 compared to APU-2 (peaking at 18 h for *EMWEY 23530* and 0 h for *EMWEY 48910*). *EMWEY 23530* and *EMWEY 48910* cDNA were cloned and expressed as polyHis-fusion proteins in *Escherichia coli*. The observed size of recombinant EMWEY 23530 was 24 kDa; the observed size of recombinant EMWEY 48910 was 35 kDa, which are consistent with the predicted size based on the coding sequences. Immunostaining 2D gel blots of *E. maxima* APU1 and APU2 oocyst/sporocyst protein with antisera specific for EMWEY 23530 identified a 33.5 kDa protein with a pH 7.4 isoelectric point (Emax p33.5). Similar 2D gel blot analysis with EMWEY 48910 identified a 41 kDa protein with a pH 7.2 isoelectric point (Emax p41). The intensity of Emax p33.5 and Emax p41 was noticeably greater in oocyst/sporocyst proteins from *E. maxima* APU1 compared to *E. maxima* APU2. This was corroborated by ELISA wherein equal amounts of total *E. maxima* APU1 and APU2 protein were probed with serial dilutions of anti-rEmax p33.5 or anti-rEmax p41. Immunofluorescence (IFA) staining of permeabilized unsporulated *E. maxima* APU1 and APU2 oocysts revealed Emax p33.5 to be localized in one end of oocysts, while Emax p41 appeared on the surface of oocysts. After sporulation, the p33.5 and p41 antigens appeared loosely associated with sporocysts. Taken together, these data confirm excess expression of two proteins in the *E. maxima* strain characterized by greater fecundity and virulence, and may provide insight into basis for phenotypic differences among different *E. maxima*.

## Introduction

Avian coccidiosis is an intestinal disease that occurs world-wide in poultry causing annual losses in excess of $ 13 billion (1). The losses stem from poor weight gain and feed conversion efficiency in infected chickens and from increased mortalities due to necrotic enteritis (NE) of which coccidiosis is a major predisposing factor. The causative organisms are protozoa in the genus *Eimeria* that are transmitted through a fecal-oral route of infection arising from the ingestion of an environmentally-resistant oocyst stage that is present in litter. Although coccidiosis in chickens can be caused by any of 7 *Eimeria*, the most problematic is *Eimeria maxima* because it infects a region of the gut that is a critical region for nutrient uptake. Moreover, *E. maxima* disrupts the intestinal epithelium and thereby is predisposing to invasion and subsequent toxin release by *Clostridium perfringens* leading to NE. In our research, two strains of *E. maxima*, namely *E. maxima* APU1 and APU2, were characterized as having different levels of pathogenicity due in part to differences in fecundity, with *E. maxima* APU1 producing greater numbers of oocysts than *E. maxima* APU2 (2). The genetic basis for these phenotypic differences has been explored using RNA-seq. This technology has been used to study *Eimeria* oocyst sporulation (3–5), to compare different *Eimeria* life cycle stages *in vivo* and *in vitro* (6–11), to compare precocious to virulent *Eimeria* (12–14), and to compare drug-sensitive to drug-resistant isolates (15–23). All of these studies have identified genes that may encode proteins involved in parasite development and drug resistance. In our research, RNA-seq analysis identified several genes whose transcripts were upregulated in the more fecund and virulent *E. maxima* APU1 (24). The present study describes the molecular characteristics of these two upregulated transcripts.

## Materials and methods

### Parasites and preparation of native protein

*Eimeria maxima* APU1 and APU2 were isolated from commercial broiler farms over 10 years ago and have been propagated every 3 mo. since isolation in susceptible chickens. Purity of the isolates was confirmed after each propagation by microscopy and ITS1-PCR (25). *Eimeria maxima* APU1 and APU2 oocysts were treated for 30 min with 6.5% sodium hypochlorite to remove contaminating bacteria, washed 5 times with diH_2_O with centrifugation at 1850 g for 10 min and suspended in Saline A (140 mM NaCl, 5 mM KCl, 4.2 mM NaHCO_3_, 0.1% glucose, pH 7.0). The oocysts were ground in a glass mortar with a Teflon pestle 75 times to release sporocysts. An aliquot of the sporocysts were subjected to *in vitro* excystation at 41°C by exposure to 0.5% trypsin and 4% sodium taurocholate (Sigma) plus 1 mM dithiothreitol (DTT) for about 45 min. Released sporozoites, intact sporocysts, and oocysts were pelleted by centrifugation for 10 min at 13,800 *g*, followed by resuspension in PBS [for IFA (see below)] or 8 M urea, 2% CHAPS, and 50 mM DTT for isoelectric focusing (see below).

### Quantitative RT-PCR

In the previous study detailing RNA-seq in *E. maxima* strains APU-1 and APU-2, oocysts during a sporulation time course were collected every 6–12 h up to 48 h (24). RNA and cDNA from oocysts of that study were used in the current study for quantitative reverse transcriptase PCR. Here, we measured transcription of genes *EMWEY 23530* (encodes 18 kDa cyclophilin) and *EMWEY 48910* (encodes a hypothetical protein) following similar procedures. In brief, RT-qPCR reactions were prepared using SsoAdvanced Universal SYBR Green Supermix (Bio-Rad, Hercules, CA, United States), with 400 nM of each primer (Table 1), and 1 μL of diluted cDNA in a total volume of 10 μL. *EMWEY 42350* (Beta tubulin, 200 nM) was used as a reference to normalize the expression levels of target genes. RT-qPCR consisted of an initial denaturing step at 95°C for 30 s, followed by 35 cycles of 95°C for 15 s, 55°C for 20 s. Melt-curve analysis entailed 55–95°C in increments of 0.5°C for 5 s. The abundance of mRNA at time points T0, T18, T36 was compared between strains APU-1 and APU-2 in triplicate reactions. Gene expression was estimated for the reference and target genes after averaging the *C*q values for each replicate at each time point. The fold change in expression was calculated using an efficiency-corrected relative expression method (26). Each experiment was performed three times, and the mean of expression change (log_2_ fold change (FC)) was calculated for each gene and time point. All primers were designed using NCBI Primer-BLAST (27) and synthesized by Integrated DNA Technologies (Coralville, IA, United States).

**Table 1:** Oligonucleotide primers used in quantitative RT-PCR analysis of RNA from *Eimeria maxima* APU1 and APU2 during oocyst sporulation.

Primer name	Target	Sequence (5′–3′)	Amplicon size (bp)
EMWEY 23530-F2	Cyclophilin	TGGTTCAGGGAGGGGATGTA	142
EMWEY 23530-R2		TTCGTGTTTGGACCGGCATT	
EMWEY 48910-F2	Hypothetical Protein	GTTGAAGAACAGCCATTCGGC	177
EMWEY 48910-R2		AGTTGTTTCCTGGTCTCCACTC	
EMWEY 43250-F2	Beta Tubulin	CACTGGTACACCGGGGAAG	102
EMWEY 43250-R2		GGTGGCATCCTGGTACTGC	

### Expression cloning

*EMWEY 23530* and *EMWEY 48910* sequences were identified in a previous RNA-seq analysis to be upregulated at all time-points during sporulation of *E. maxima* oocysts (24). Further comparative transcriptome analysis between two strains of *E. maxima* (APU1 and APU2) revealed about a two-fold greater number of *EMWEY 23530* and *EMWEY 48910* transcripts in *E. maxima* APU1 than in *E. maxima* APU2. The open reading frame of *EMWEY 23530* and *EMWEY 48910* were synthesized by a commercial company (GenScript, Piscataway NJ) containing 5’ XhoI and 3’ EcoRI sites for cloning into pBluescript II SK. The cDNA inserts were excised with XhoI and EcoRI, cloned into pTrcHisA (Invitrogen) with DNA ligase, and recombinant plasmid used to transform *Escherichia coli* BL21. Recombinant clones were expanded in LB-Amp at 37°C and induced at log phase growth (O.D._600_ = 0.5) for 4 h with 1 mM IPTG. The induced cells were harvested by centrifugation at 1850 RPM for 10 min. The cell pellets were extracted first for 30 min with NBB containing 0.1 mg/mL lysozyme and protease inhibitors, followed by DNase and RNase treatment for an additional 30 min on a rocker. The soluble fraction after NBB treatment was collected by centrifugation, and the resulting pellet was further extracted with DBB for 30 min on a rocker, followed by centrifugation to retrieve denaturing soluble (DS) supernatant. Recombinant EMWEY 23530 and EMWEY 48910 proteins were purified by affinity chromatography using NiNTA agarose (Invitrogen) and NiNTA eluates were analyzed by SDS-PAGE and immunoblotting (see below).

### Preparation of polyclonal anti-EMWEY 23530 and anti-EMWEY 48910 sera

Eluates from NiNTA-column purification of recombinant EMWEY 23530 (rEMWEY 23530) and EMWEY 48910 (rEMWEY48910) were used to immunize New Zealand White rabbits (2/recombinant antigen) (Pacific Immunology, Ramona CA). Immunizations utilized Freund’s Complete Adjuvant in the primary immunization and Freund’s Incomplete Adjuvant in booster immunizations at 3, 6, and 10 weeks post-primary immunization. Blood was collected prior to primary immunization, at various time-points during and after the final booster immunization, and processed for serum using standard procedures (28).

### Isoelectric focusing

*Eimeria maxima* APU1 and APU2 oocyst, sporocyst, and sporozoite protein from 10^7^ oocysts were extracted with 8M urea, 2% CHAPS, and 50 mM DTT at RT for 1 h on a rocker, followed by centrifugation for 10 min at 13,800 *g*. The supernatant protein concentration was estimated by BCA assay. *Eimeria maxima* APU1 or APU2 protein (100 μg) was mixed with Rehydration/Sample buffer (Bio-Rad, Hercules CA) and adsorbed to ReadyStrip IPG strips pH 7–10 for 10 h at RT following manufacturer’s instructions (Bio-Rad). The IPG strips were then subjected to isoelectric focusing in the following steps: Step 1–250 v, 20 min, linear ramp speed; Step 2–4,000 v, 2 h, linear ramp speed; Step 3–4,000 v to reach 10,000 v-hr, rapid ramp speed. After IEF, the IPG strips were rinsed with Equilibration Buffers I and II for 10 min each, then inserted into a preparative well of SDS-PAGE and overlain with low melting point agarose containing trace amounts of Bromophenol Blue.

### SDS-PAGE/immunoblotting

Unpurified and NiNTA-purified recombinant EMWEY 23530 (rEMWEY 23530) and EMWEY 48910 (rEMWEY 48910) proteins were analyzed by SDS-PAGE followed by transblotting to Immobilon membrane (Millipore-Sigma Burlington MA), followed by immunostaining with mouse anti-His antibodies (Invitrogen-Thermo Scientific, Waltham MA) using standard procedures (29). For 2D gel analysis, IPG strips were subjected to SDS-PAGE for fractionation of IEF-resolved native *E. maxima* APU1 and APU 2 proteins followed by transblotting to Immobilon membrane. The membranes were immunostained with rabbit anti-rEMWEY 23530 or anti-rEMWEY 48910 sera using standard procedures (29).

### Enzyme-linked immunosorbent assay

*Eimeria maxima* APU1 and APU2 native protein extracted for 2D gel immunoblotting was diluted in carbonate buffer (pH 9.5) and adsorbed for 6 h at 37°C onto wells of Immulon II microtiter plates (Thermo Scientific) at 1.0 μg/well. The wells were washed with PBS-containing 0.05% Tween 20 (BSA-Tw), blocked for 30 min with 1% BSA in PBS-Tw, incubated for 1 h at RT with serial dilutions of rabbit anti-EMWEY 23530 or anti-EMWEY 48910 sera or pre-immunization sera, followed by alkaline-phosphatase labeled anti-rabbit IgG (Sigma, 1:1000 dilution) for an additional 1 h at RT. Binding was visualized by addition of alkaline-phosphatase substrate (1 mg/mL p-nitrophenyl phosphate, disodium) and measured on a microtiter plate reader (SpectraMax 190, Molecular Devices, San Jose, CA) at 405 nm. Antibodies were removed after each step by 3 washes with PBS-Tw.

### Immunofluorescence assay

*Eimeria maxima* APU1 and APU2 oocysts, sporocysts, and sporozoites (see above) were adhered to the surface of 8-well microscope slides (MP Biomedicals, San Diego CA), air-dried, and then treated with cold methanol for 5 min, followed by a brief wash with PBS-Tw. The wells were first treated with PBS-Tw + 1% BSA, followed by a 1 h incubation with a 1:100 or 1:500 dilution of rabbit anti-EMWEY 23530 or anti-EMWEY 48910 sera or preimmunization sera, followed by a 1 h incubation with a 1:100 dilution of FITC-labeled goat anti-rabbit IgG (Sigma). The slides were washed between each incubation step with PBS-Tw, allowed to air dry after the last wash, overlaid with VectaShield (Vector Laboratories, Newark NJ) anti-bleaching medium and a coverslip. The slides were examined on a Zeiss microscope and images captured using software.

## Results and discussion

### Quantitative RT-PCR

**Figure 1:**
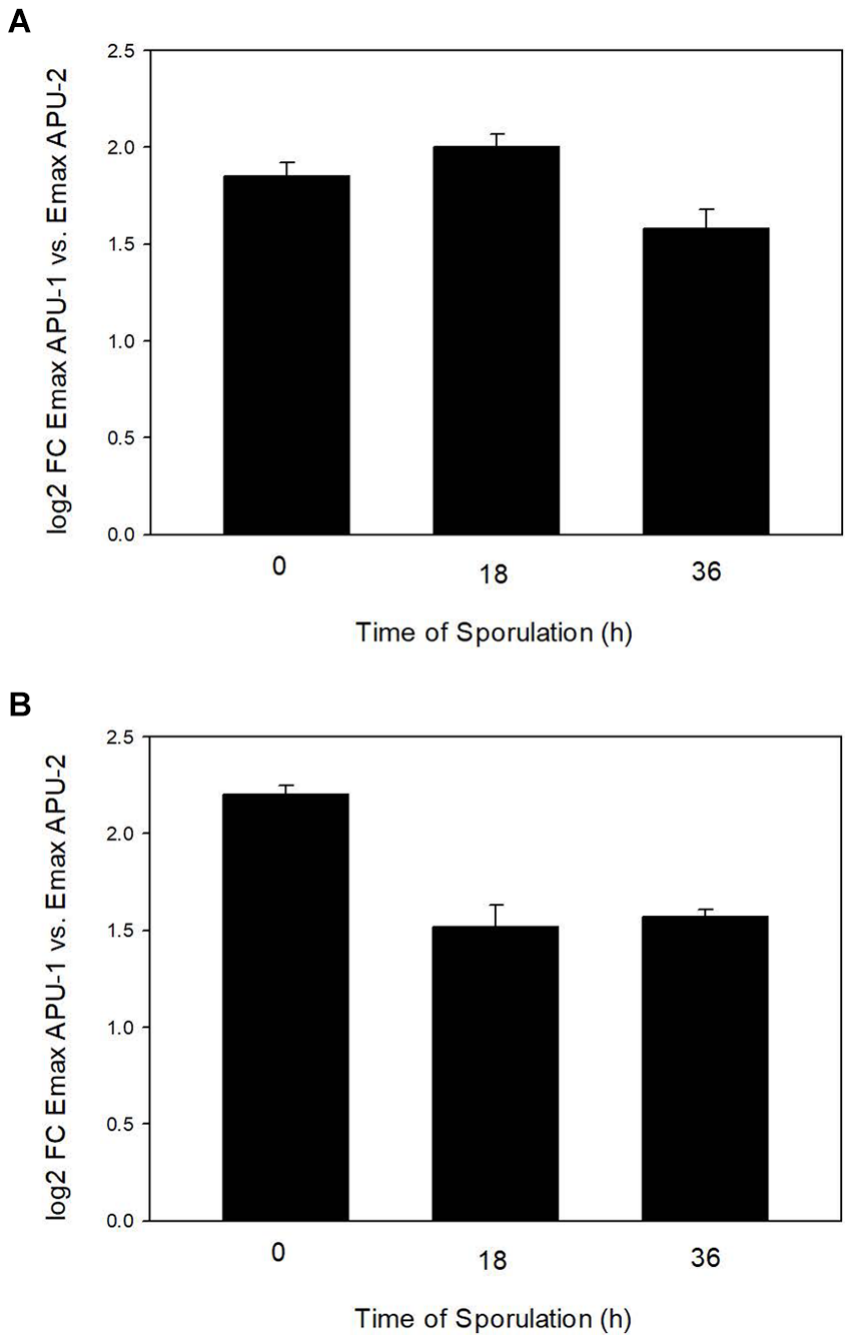
Comparison of increased *EMWEY 23530* **(A)** and *EMWEY 48910* **(B)** transcript levels in *Eimeria maxima* APU1 vs. *E. maxima* APU2 at different times of sporulation as measured by quantitative RT-PCR analysis.

qRT-PCR using primers directed to either *EMWEY 23530* or *EMWEY 48910* corroborated our previous RNA-seq findings in that both *EMWEY 23530* and *EMWEY 48910* were transcribed 1.5–2.0 log_2_ higher at all timepoints in *E. maxima* APU1 compared to *E. maxima* APU2 (Figures 1A,B). In the current study, the greatest difference in *EMWEY 23530* expression between *E. maxima* APU1 and APU2 was at 18 h sporulation (Figure 1A); for *EMWEY 48910* was at 0 h sporulation (Figure 1B). These data along with the IFA staining pattern suggest that *EMWEY 23530* and *EMWEY 48910* code for proteins that play some role in oocyst wall formation.

### Expression of recombinant protein and identification of relevant native protein

**Figure 2:**
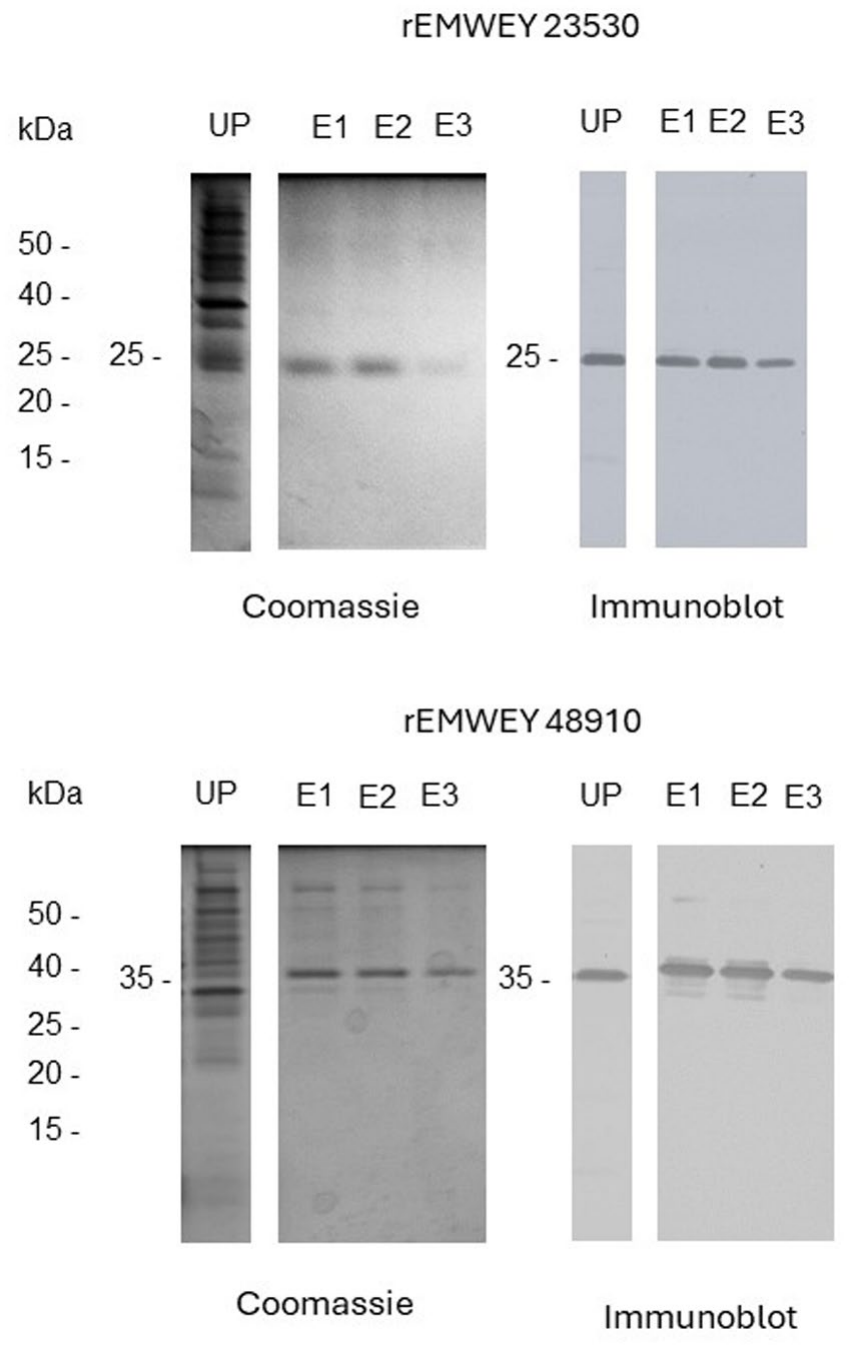
SDS-PAGE analysis of unpurified (UP) and NiNTA affinity chromatography-purified recombinant EMWEY 23530 (upper panel) and recombinant rEMWEY 48910 protein (lower panel) visualized by Coomassie Blue staining (left panels) or immunoblotting with mouse anti-His tag sera (right panels). UP-unpurified, E1-, E2-, E3-NiNTA eluate fractions used in generation of rabbit antisera, kDa-relative molecular size markers.

**Figure 3:**
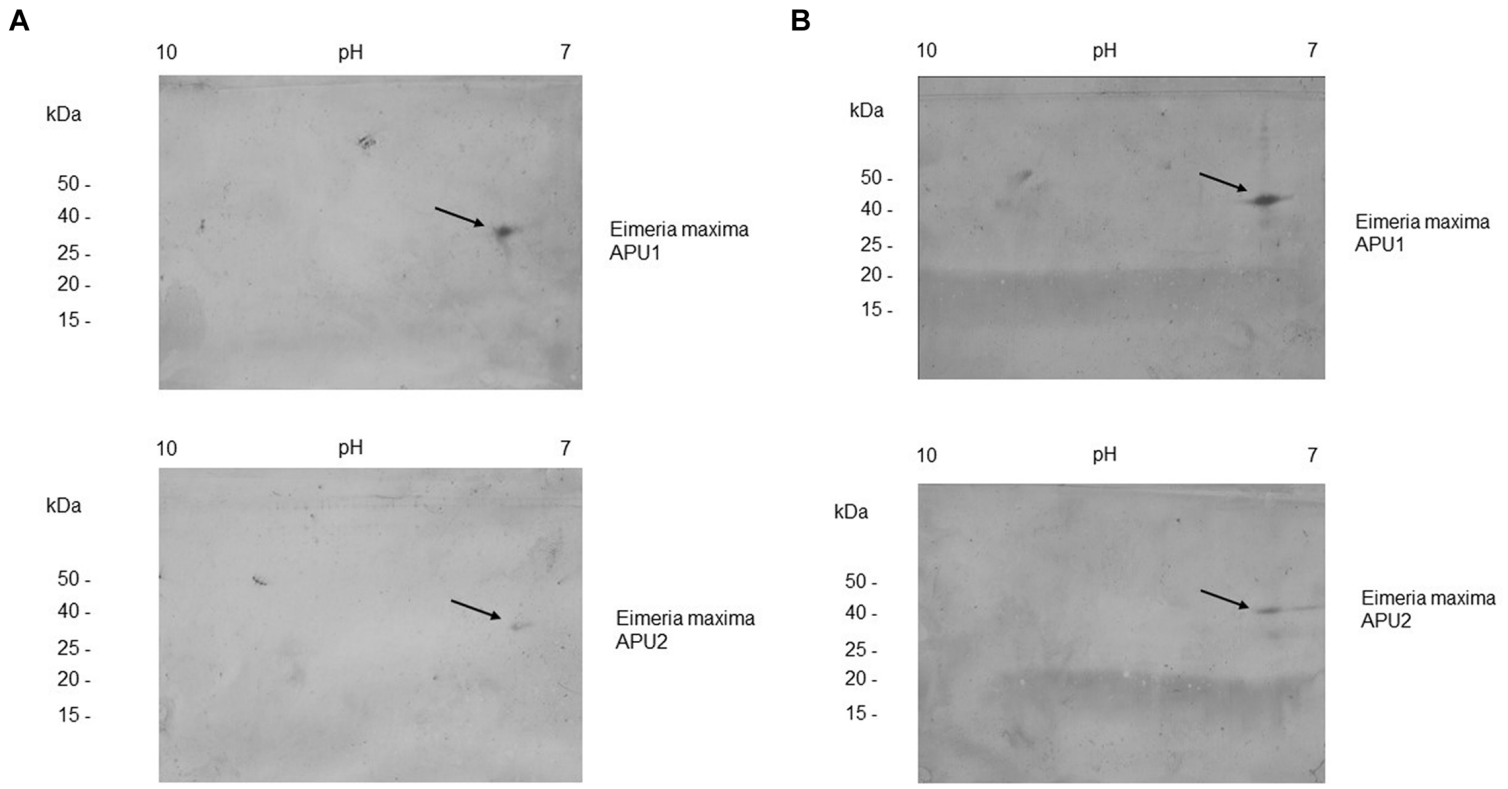
2D gel immunoblot analysis of *Eimeria maxima* APU1 or *E. maxima* APU2 total oocyst, sporocysts, sporozoite protein resolved by isoelectric focusing (IEF) in the first dimension and SDS-PAGE in the second dimension. Equal amounts of total *E. maxima* APU1 and APU2 protein were subjected to 2D gel electrophoresis and immunostained with antisera specific for recombinant EMWEY 23530 **(A)**, or EMWEY 48910 **(B)**.

Expression cloning of *EMWEY 23530* and *EMWEY 48910* as polyHis fusion proteins in *Es. coli* revealed by SDS-PAGE/immunoblotting a 24–26 kDa protein for the former and a 35 kDa protein for the latter (Figure 2). The predicted size of recombinant EMWEY 23530 based on the DNA sequence is 20.5 kDa which with the addition of the upstream polyHis tail (~5 kDa) would give rise to the observed 24–26 kDa protein. The observed (35 kDa) and predicted (29.5 kDa) sizes of recombinant EMWEY 48910 is also in agreement with the DNA sequence. Employing 2D gel blots of native *E. maxima* protein, polyclonal antisera specific for EMWEY23530 identified a native 33.5 kDa protein with a pI = 7.4 (Figure 3). A similar method using antisera specific for EMWEY 48910 identified a 41 kDa protein with a pI = 7.2 (Figure 3). The difference between observed (33.5) and predicted (20.4) sizes of EMWEY 23530 may be due secondary processing such as glycosylation and to 3D folding of the protein. A similar size discrepancy between observed (41 kDa) and predicted (29.5) EMWEY 48910. Software for predicting *N*-glycosylation sites^1^ and *O*-glycosylation sites^2^ found 2 potential *N*-glycosylation sites and 8 potential *O*-glycosylation sites in EMWEY 23530. Similar analyses for EMWEY 48910 found 2 potential *N*-glycosylation sites and 5 potential *O*-glycosylation sites.

**Figure 4:**
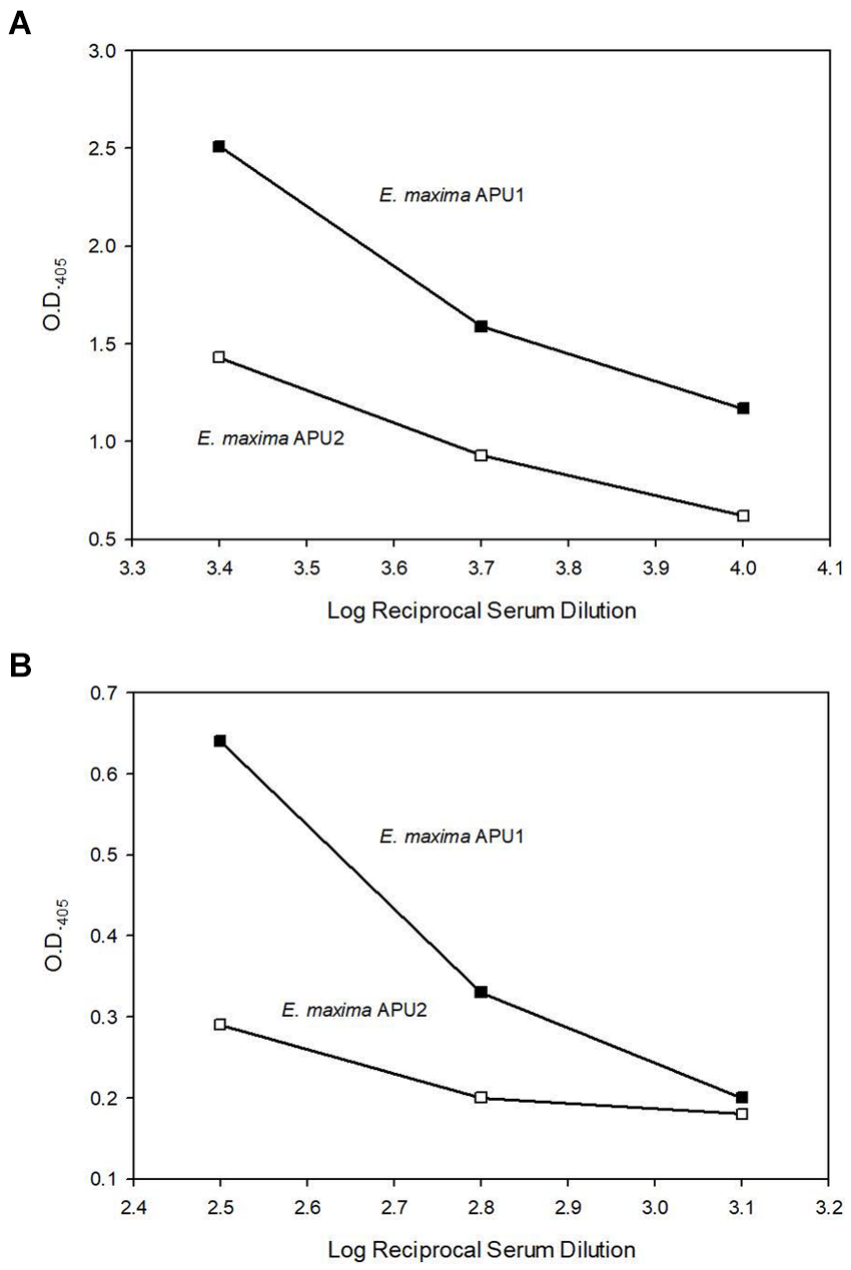
Enzyme-linked immunosorbent assay (ELISA) analysis of equal amounts (1 μg) of total *Eimeria maxima* APU1 (closed symbols) or *E. maxima* APU2 (open symbols) protein adhered to Immulon 2 HB plates and probed with serial dilutions of anti-EMWEY 23530 sera **(A)** (log reciprocal dilutions 3.4, 3.7, and 4.0) or anti-EMWEY 48910 sera **(B)** (log reciprocal dilutions 2.5, 2.8, and 3.1).

The higher transcript levels of *EMWEY 23530* and *EMWEY 48910* in *E. maxima* APU1 compared to *E. maxima* APU2 was also observed at the protein level as observed in 2D immunoblots of *E. maxima* APU1 or *E. maxima* APU2 native oocyst/sporocyst protein. *Eimeria maxima* APU1 displayed greater amounts of the native 33.5 (Figure 3A) and 41 kDa proteins (Figure 3B) than *E. maxima* APU2. The greater expression of native EMWEY 23530 and EMWEY 48910 was corroborated by ELISA using total native *E. maxima* APU1 and APU2 protein probed with anti-rEMWEY 23530 or anti-rEMWEY 48910 sera (Figure 4). These data suggest that higher *EMWEY 23530* and *EMWEY 48910* transcript levels leads to greater amounts of Emax p33.5 and Emax p41 protein in *E. maxima* APU1 compared to *E. maxima* APU2.

### Antigen localization by IFA

**Figure 5:**
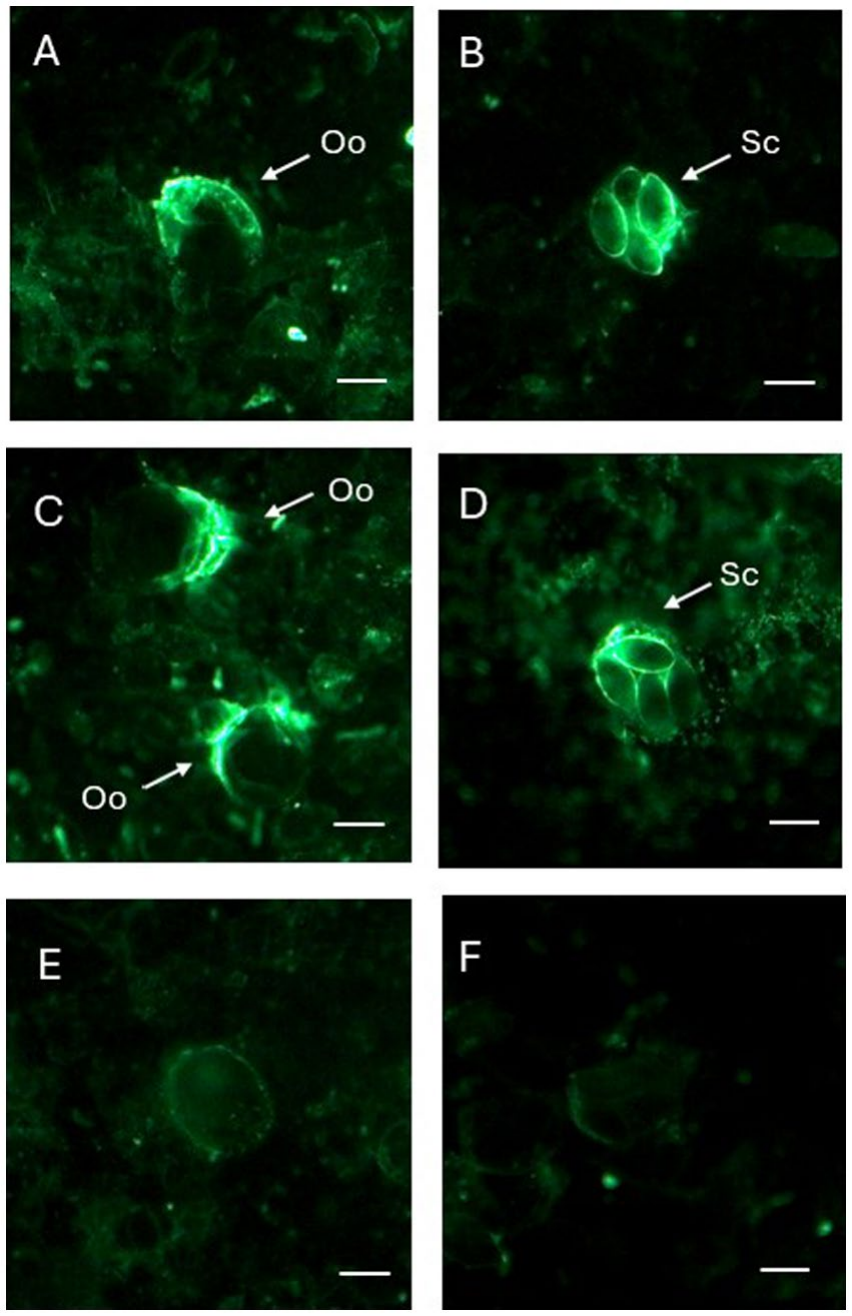
Immunofluorescence (IFA) staining of *Eimeria maxima* APU1 **(A,B,E)** or *E. maxima* APU2 **(C,D,F)** with anti-recombinant EMWEY 23530 **(A,C)** or anti-recombinant EMWEY 48910 **(B,E)** sera. Oo, oocysts; Sc, sporocysts. Preimmunization control IFA staining **(E,F)**. Bar = 10 μm.

EMWEY 23530 appeared to localize to antigens found internal and external to *E. maxima* APU1 and APU2 oocysts whereas EMWEY 48910 was only found inside oocysts and appeared to be associated with sporocysts (Figure 5). The reactivity of antisera, particularly against rEMWEY48910, to sporocysts inside oocysts may be explained by the variability in morphology of oocysts after grinding and excystation. Many oocysts and sporocysts did not react with the antisera (data not shown) suggesting that the antigen is present inside oocysts and is released when the oocyst wall is completely broken open. No internal labeling of sporozoites was observed with anti-EMWEY 48910 sera and no difference in the staining pattern between *E. maxima* APU1 and APU2 oocysts, sporocysts, and sporozoites was found (data not shown). The similarity in IFA staining of *E. maxima* APU1 and APU2 which is different than the increased binding as observed by 2D gel immunoblotting and ELISA probably reflects the more random nature of microscopy staining. 2D immunoblots and ELISA utilize total protein which would be less susceptible to effects on morphology.

## Conclusion

Previous studies using RNAseq to compare RNA transcripts between two different strains of *E. maxima* during sporulation identified a number of upregulated genes in *E. maxima* APU1 compared to *E. maxima* APU2. This study employed qRT-PCR to confirm the relatively higher levels of *EMWEY 23530* and *EMWEY 48910* transcripts in *E. maxima* APU1. This greater expression was reflected in greater intensity of native Emax p33.5 and Emax 41 protein as observed in 2D gel immunoblots and in ELISA using antisera to recombinant EMWEY 23530 and EMWEY 48910. The former appears to be associated with an antigen inside and outside sporulated oocysts, whereas the latter is only found inside oocysts and loosely associated with sporocysts. Whether these proteins have any role in the differences in fecundity between *E. maxima* APU1 and APU2 remains unknown.

## Data Availability

The raw data supporting the conclusions of this article will be made available by the authors, without undue reservation.

## Footnotes

^1^https://services.healthtech.dtu.dk/services/NetNGlyc-1.0/

^2^https://services.healthtech.dtu.dk/services/NetOGlyc-4.0/
